# Assessment of the Capability of Artificial Intelligence for Psychiatric Diagnosis

**DOI:** 10.1192/j.eurpsy.2024.1722

**Published:** 2024-08-27

**Authors:** N. Laherrán, R. Palacios, A. Vázquez

**Affiliations:** Psychiatry, Jerez de la Frontera University Hospital, Jerez De La Frontera, Spain

## Abstract

**Introduction:**

Psychopathological exploration (PPE) involves an assessment of the mental state of patients, where psychological signs and symptoms are analyzed, which collectively form a syndrome. To conduct this assessment, the clinician must utilize their expertise to identify the presence and authenticity of a series of symptoms that, once recognized, allow for a diagnosis (1). The presence of this subjective component could explain why, despite the continuous growth of artificial intelligence (AI), its application in clinical psychiatry practice remains limited. However, the combination of the clinician's work with AI could enhance diagnostic accuracy and our understanding of diseases (2).

**Objectives:**

The objective of this study is to investigate whether AI can make accurate diagnoses through an initial psychopathological evaluation.

**Methods:**

A random sample was selected from our medical records of all patients admitted to our acute mental health inpatient unit through the hospital's emergency services in the year 2022. An anonymized database was created, including sociodemographic information, the results of the psychopathological assessment in the emergency department, and the diagnosis at the time of discharge. The psychopathological assessment conducted in the emergency department was provided to the AI chatbot ChatGPT, with a request to establish a diagnosis according to the DSM-5. Diagnoses such as brief psychotic disorder, schizophreniform disorder, and schizophrenia were considered, given their acute symptom similarities, as well as major depressive disorder (unipolar) and bipolar disorder. The level of agreement between both diagnoses was evaluated using the kappa coefficient.

**Results:**

The sample consisted of 15 patients, of whom 60% were male, with a mean age of 45 years (standard deviation = 15.6). 73.3% of the patients had prior mental health follow-up, and 66.7% had been previously hospitalized. Diagnoses included psychotic disorder in 33.3% of cases, bipolar disorder with manic episode in 26.7%, depressive disorder in 13.3%, delusional disorder in 13.3%, schizoaffective disorder in 6.7%, and borderline personality disorder in 6.7%. A kappa value of 0.561 was obtained, indicating a moderate degree of agreement between the diagnoses.

**Conclusions:**

Despite the inherent subjectivity in psychopathological assessment, this study suggests that AI, in the form of natural language processing chatbots like ChatGPT, can be a useful tool to assist mental health professionals in the diagnostic process. While AI shows promising potential, it should not entirely replace the experience and clinical judgment of mental health professionals. Instead, the importance of potential collaboration between AI and clinicians for achieving more precise and comprehensive diagnoses is highlighted.

**Disclosure of Interest:**

None Declared

